# Fluorometric and Colorimetric Method for SARS-CoV-2 Detection Using Designed Aptamer Display Particles

**DOI:** 10.3390/bios14030113

**Published:** 2024-02-20

**Authors:** Ki Sung Park, Anna Choi, Tae-In Park, Seung Pil Pack

**Affiliations:** Department of Biotechnology and Bioinformatics, Korea University, Sejong 30019, Republic of Korea; pks850528@korea.ac.kr (K.S.P.); dkssk0714@korea.ac.kr (A.C.); jjuft@korea.ac.kr (T.-I.P.)

**Keywords:** SARS-CoV-2 aptamer, SARS-CoV-2 spike protein-binding aptamer, particle display, aptamer display particles, fluorometric aptasensor, colorimetric aptasensor

## Abstract

SARS-CoV-2, the virus responsible for the COVID-19 pandemic, has spurred the urgent need for practical diagnostics with high sensitivity and selectivity. Although advanced diagnostic tools have emerged to efficiently control pandemics, they still have costly limitations owing to their reliance on antibodies or enzymes and require high-tech equipment. Therefore, there is still a need to develop rapid and low-cost diagnostics with high sensitivity and selectivity. In this study, we generated aptamer display particles (AdP), enabling easy fabrication of a SARS-CoV-2 detection matrix through particle PCR, and applied it to diagnosis using fluorometric and colorimetric assays. We designed two AdPs, C1-AdP and C4-AdP, displayed with SpS1-C1 and SpS1-C4 aptamers, respectively, and showed their high binding ability against SARS-CoV-2 spike protein with a concentration-dependent fluorescence increase. This enabled detection even at low concentrations (0.5 nM). To validate its use as a diagnostic tool for SARS-CoV-2, we designed a sandwich-type assay using two AdPs and high-quality aptamers targeting SARS-CoV-2 pseudoviruses. The fluorometric assay achieved a detection limit of 3.9 × 10^3^ pseudoviruses/mL. The colorimetric assay using an amplification approach exhibited higher sensitivity, with a detection limit of 1 × 10^1^ pseudoviruses/mL, and a broad range of over four orders of magnitude was observed.

## 1. Introduction

Coronaviruses (CoVs) are a highly diverse family of enveloped, positive-sense, single-stranded RNA viruses of both medical and veterinary importance [1,2]. CoVs cause various diseases with varying degrees of severity that affect the respiratory, enteric, hepatic, and neurological systems in both humans and animals [1,3]. To date, seven human coronaviruses (HCoVs) have been identified. Four of these HCoVs (HCoV-NL63, HCoV-HKU1, HCoV-OC43, and HCoV-229E) cause asymptomatic or mild respiratory infections [3,4]. In 2002 and 2012, two novel lethal HCoVs, severe acute respiratory syndrome (SARS)-CoV (SARS-CoV) and Middle East respiratory syndrome (MERS)-CoV (MERS-CoV), emerged as highly pathogenic, causing severe diseases [5]. The seventh member of the CoV family that infects humans, SARS-CoV-2, emerged in late December 2019 and has caused an ongoing global pandemic with high morbidity and mortality, posing a severe threat to public health and the global economy [6,7,8].

The most effective way to control pandemics such as COVID-19 is to inhibit viral spread through the early diagnosis of patients with suspected infections and screening [9]. At the very early stages of the COVID-19 pandemic, a pan-CoV reverse transcription-polymerase chain reaction (pan-CoV RT-PCR) assay was employed for the early detection of infections, which allowed the detection of all the abovementioned known HCoVs, except SARS-CoV-2 [10]. After updating the sequences of the SARS-CoV-2 genome, reverse transcription quantitative polymerase chain reaction (RT-qPCR)-based diagnosis has become the gold standard for early detection [11,12]. However, these RT-qPCR-based molecular diagnostics require a turnaround time of >24 h to screen and diagnose patients with suspected SARS-CoV-2 infection. Over the past three years, numerous diagnostic approaches, such as lateral flow-based immunoassays [13] and CRISPR-based assays (e.g., SHERLOCK [14] and DETECTR [15]), have been developed. Although these assays reduce the time required for diagnosis, they incur high production costs owing to their reliance on antibodies or enzymes. In addition, rapid antigen tests that are broadly used currently have the limitation of a high false-positive rate [16]. Therefore, there is a need to develop rapid and low-cost diagnostics with high sensitivity and selectivity.

Over the last three decades, nucleic acid aptamers have garnered substantial interest as versatile molecular recognition elements (MREs) and have proven to be potential alternatives to antibodies [17,18,19,20]. These aptamers, often known as “chemical antibodies” [21], have been utilized with antigen detection as MREs, and have high affinity and specificity to various types of target molecules [22]. Compared with the monoclonal antibody, the aptamer has numerous advantages, such that it can be chemically synthesized in a cost-effective manner and is stable under thermal and pH conditions because it is an oligonucleotide, not a protein, and allows for rapid screening by in vitro evolution. The most powerful feature is that the target is not limited to macromolecules, such as proteins and whole cells, but also small molecules, such as metal ions and toxins [21,23].

By utilizing these features of aptamers, several DNA aptamer-based analytical approaches, such as the aptamer-based lateral flow assay [24] and the enzyme-linked oligonucleotide assay (ELONA), have been thoroughly developed to replace antibody-based analytical methods [25]. Furthermore, highly sensitive detection methods for SARS-CoV-2 have been developed by incorporating aptamers into various advanced platforms and have exhibited excellent accuracy [26,27]. However, these advanced technologies not only make it difficult to conjugate aptamers, fabricate substrates, and interpret the resulting data, but also require high-cost equipment for measurement. In biosensors that use affinity reagents, binding affinity is the main factor determining the dynamic range of the sensing platform [28,29,30]. Therefore, sufficient functionality for sensitive detection relies primarily on the quality of the aptamer, and the detection limit depends on the affinity of the aptamer. We focused on high-quality aptamers that provided sufficiently reliable signals and accuracy for diagnostics, even without advanced platforms.

In a previous study, we successfully discovered two novel DNA aptamers against SARS-CoV-2 spike protein using only four rounds of particle display [31]. These DNA aptamers, designated as SpS1-C1 and SpS1-C4, against the SARS-CoV-2 spike protein exhibited high affinity with Kd values of below 2 nM and showed a high-quality feature of universal binding to the spike protein of SARS-CoV-2 variants with excellent accuracy.

**Figure 1:**
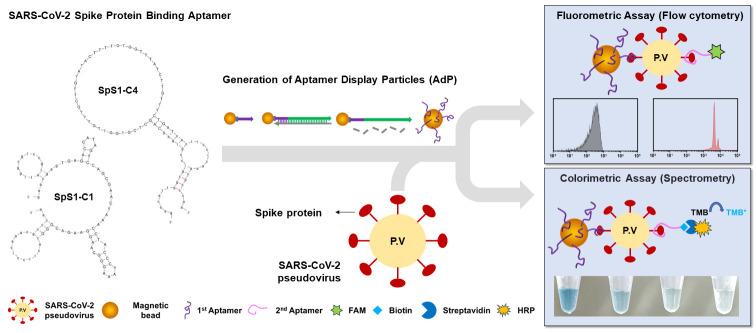
Schematic illustration of AdP-based SARS-CoV-2 detection assays. Two novel DNA aptamers, SpS1-C1 and SpS1-C4, for SARS-CoV-2 spike protein were isolated by particle display selection. The secondary structures of these aptamers were predicted using mFold. Each of the spike protein-binding AdPs was generated by particle PCR on the FP-coated magnetic bead. Utilizing these SpS1-AdPs, we designed a sandwich-type SARS-CoV-2 pseudovirus assay involving fluorometric and colorimetric assays. C1-AdP and C4-AdP were employed as primary capture probes, and SpS1-C1 and SpS1-C4 aptamers were used as secondary detection probes. FAM and biotin modification were introduced at the 5′ end of the secondary aptamer for fluorometric and colorimetric assays, respectively.

Using these high-quality aptamers, in this study, we generated aptamer display particles (AdP), enabling easy fabrication of the SARS-CoV-2 detection matrix through particle PCR, and applied it to diagnosis using fluorometric and colorimetric assays (Figure 1). We focused on generalizing the detection method for SARS-CoV-2 using our spike protein-binding AdPs (SpS1-AdPs) for a simple analysis using flow cytometry or spectrometry. The aptamer-functionalized micrometer-sized magnetic particles enabled us to perform better and more selective washing and recovery, allowing the precise analysis of individual particles. The binding performance of our SpS1-AdPs was evaluated using flow cytometry with various concentrations of recombinant trimeric spike proteins. High binding ability was observed even at low concentrations (0.5 nM). Moreover, we designed a sandwich-type approach to improve the selectivity and sensitivity of SpS1-AdPs as primary capture probes and the signal-labeled aptamers SpS1-C1 or SpS1-C4 as secondary detection probes. We used a SARS-CoV-2 pseudovirus to assess the performance of our AdP-based sandwich-type sensing system for the diagnosis approach of SARS-CoV-2. The fluorometric assay showed a detection limit of 3.9 × 10^3^ pseudoviral particles/mL. The colorimetric assay with signal amplification achieved high sensitivity, with a detection limit of 1 × 10^1^ pseudoviral particles/mL and a broad range of over four orders of magnitude (10^5^–10^1^ pseudoviral particles/mL). Our high-quality SpS1-AdPs achieved sensitive detection of SARS-CoV-2, even without advanced platforms.

## 2. Materials and Methods

### 2.1. Chemicals and Materials

Dynabeads^TM^ MyOne^TM^ Carboxylic Acid (1-μm size) and Streptavidin C1 (1-μm size) were purchased from Invitrogen (Carlsbad, CA, USA), and 1-ethyl-3-(3-dimethylaminopropyl)-carbodiimide (EDC), EZ-Link™ Biotinylation Kit, Alexa Fluor™ 488-conjugated Streptavidin, and 1-Step™ TMB ELISA Substrate Solutions were purchased from Thermo Fisher Scientific (Waltham, MA, USA). N-hydroxysulfosuccinimide (sulfo-NHS) and Tween-20 were purchased from Sigma-Aldrich (St. Louis, MO, USA). GoTaq^®^ G2 Colorless Master Mix was purchased from Promega (Madison, WI, USA). Dulbecco’s phosphate-buffered saline (DPBS) was purchased from Biowest (Nuaillé, France). The recombinant SARS-CoV-2 spike protein extracellular domain (ECD) (ref 40589-V08H4) was purchased from Sino Biological (Beijing, China). COVID-19 Spike 614G Coronavirus Pseudovirus (MBS434278) was purchased from MyBioSource (San Diego, CA, USA). VECTASTAIN Elite ABC HRP Kit (PK-6100) was purchased from Vector Laboratories (Burlingame, CA, USA).

### 2.2. Oligonucleotides

The single-stranded DNA (ssDNA), including aptamers and modified primers, was synthesized by Integrated DNA Technologies (IDT; Coralville, IA, USA). All sequences described in this study are listed in Supplementary Information, Table S1.

### 2.3. Conjugation of Forward Primer (FP) to Magnetic Particles

Forward primer (FP) was synthesized with modification of 5′-Amino modifier C12 (AmM-FP). Dynabeads^TM^ MyOne^TM^ Carboxylic Acid (1 μm, 10^7^ beads/μL) were washed with 500 μL of 0.01 N sodium hydroxide (NaOH), followed by washing five times with 500 μL of ultra-pure water. The washed beads were reacted with 30 nM AmM-FP, 250 mM EDC, 1 mM imidazole chloride, and 200 mM NaCl to a total volume of 150 μL. After vortexing and sonication, the mixture was incubated overnight at 25 °C with gentle rotation. After conjugation between the beads and AmM-FP, the FP-conjugated magnetic beads (FP beads) were washed twice with 500 μL of DPBS. To block the remaining functional groups on the bead surface, the FP beads were incubated with 20 mM amino-PEG-methyl in an MES solution containing EDC/NHS and quenched with 50 mM Tris-HCl buffer (pH 8.0, with 0.05% Tween-20). Finally, the FP beads were stored in 500 μL of 10 mM Tris-HCl buffer (pH 8.0, with 0.01% Tween-20). To verify whether FP was well-conjugated to the bead surface, we prepared 2 × 10^6^ FP beads hybridized with 1 μM FAM-modified FP complementary strand in PBST buffer (0.025% Tween-20 in DPBS). The fluorescence signals of the FAM-labeled FP beads were analyzed using an Attune^TM^ NxT Flow Cytometer (Thermo Scientific, Waltham, MA, USA).

### 2.4. Generation of Aptamer Display Particles (AdP)

We generated SpS1-AdPs with SARS-CoV-2 binding aptamers, SpS1-C1 and SpS1-C4, respectively, by particle PCR. First, 3 × 10^7^ FP-coated beads were suspended in a 100 μL of particle PCR mixture comprising 1X PCR Master Mix, 25 mM MgCl_2_, 1 μM FAM-modified reverse primer (RP), and 10 nM of each aptamer template. The PCR was performed as follows: pre-denaturing at 95 °C for 3 min, followed by 25 cycles of 95 °C for 30 s, 58 °C for 30 s, and 72 °C for 45 s. After every five cycles at the 72 °C elongation step, the mixture was vortexed and sonicated to avoid the aggregation of beads. Following PCR amplification, the particles were washed with PBST. In addition, the SpS1-AdPs were washed ten times with 100 mM NaOH to generate a single strand and then four times with PBST. Finally, we measured the fluorescence signal using flow cytometry to confirm the efficiency of particle PCR.

### 2.5. Flow Cytometry Analysis of Spike Protein Binding by SpS1-Aptamer Display Particles

Recombinant SARS-CoV-2 spike protein extracellular domain (ECD) (ref 40589-V08H4) was purchased from Sino Biological (Beijing, China). The spike protein was biotinylated for fluorescence labeling. Then, the protein was incubated with a 20-fold molar excess of biotin reagent for 30 min at 25 °C and removed free biotin via Zeba Desalt Spin Column (0.5 mL, Pierce Biotechnology, Rockford, IL, USA). To confirm that biotinylation was complete on the spike protein, we used streptavidin-coated magnetic beads for immobilization. Next, we labeled it with Alexa Fluor 488-conjugated streptavidin in sandwich form and analyzed them using a flow cytometer.

A total of 1.5 × 10^6^ of SpS1-AdPs were incubated with various concentrations of biotinylated spike protein ranging from 0 to 300 nM in a total volume of 20 μL with PBSMCT binding buffer (1 mM MgCl_2_, 1mM CaCl_2_, 0.025% tween-20 in DPSB). The mixture was incubated for 1 h at 25 °C on a gentle rotation. After binding, the AdP–spike protein complex was washed three times with binding buffer and 50 nM Alexa Fluor 488-conjugated streptavidin (Invitrogen, Carlsbad, CA, USA) was added for 10 min to reduce non-specific binding. We then washed thrice with a binding buffer and analyzed the fluorescence signals using flow cytometry.

### 2.6. Fluorometric Assay for Detection of SARS-CoV-2 Pseudoviruses

The ability of SpS1-AdPs to detect the SARS-CoV-2 pseudovirus was verified using a sandwich-type fluorometric assay. SpS1-AdPs and various concentrations of pseudoviruses (diluted from 5 × 10^5^ to 1 × 10^1^ pseudoviral particles using the PBSMCT) were prepared. The SpS1-AdPs (1.5 × 10^6^ particles) were suspended in 50 μL of the diluted pseudovirus solutions and incubated for 1 h at 25 °C on a gentle rotation. After pseudovirus binding to the AdP, the complex was washed thrice with vortexing and sonication using PBSMCT and was then allowed to react with 50 μL of 10 μM FAM-labeled secondary aptamer for 1 h at 25 °C on a gentle rotation, followed by washing with PBSMCT. The fluorescence signals of the FAM-labelled aptamer-coated pseudovirus were analyzed by flow cytometry.

### 2.7. Colorimetric Assay for Detection of SARS-CoV-2 Pseudoviruses

Binding between AdP and pseudoviruses was performed under identical conditions as in the fluorometric assay. To improve the sensitivity of the assay, we introduced biotin into the secondary aptamer for signal amplification by the catalytic oxidation of TMB by horseradish peroxidase (HRP). After the formation of the AdP–pseudovirus complex, we added 10 μM biotin-modified secondary aptamer. Sufficient washing was performed using PBSMCT, and we additionally reacted the avidin and biotinylated HRP with a 10 min limitation to reduce non-specific binding. After completing a sandwich formation, 100 μL of 1-step^TM^ Ultra TMB-ELISA (Thermo Scientific, Waltham, MA, USA) was added and incubated for 10 min at 25 °C for color development, followed by the addition of 100 μL stop solution (2 M sulfuric acid). Absorbance was measured at 450 nm using a spectrometer (Tecan, Infinite^TM^ M200 Pro, Männedorf, Switzerland).

## 3. Results and Discussion

### 3.1. Fabricating of Aptamer Display Particles (AdP)

In a previous study, we successfully isolated two novel DNA aptamers against the SARS-CoV-2 spike protein via only four rounds of particle display using a high-throughput screening method [31]. These DNA aptamers, designated as SpS1-C1 and SpS1-C4, exhibited high affinity with Kd values below 2 nM (Supplementary Information, Table S2) and showed high-quality features for the universal detection of SARS-CoV-2 variants with excellent accuracy. Utilizing these aptamers, we designed aptamer display particles, referred to as AdP, allowing easy fabrication of a SARS-CoV-2 diagnostic matrix for application in sandwich-type fluorometric and colorimetric assays (Figure 1). We believe that high-quality aptamers can provide sufficiently reliable signals and accuracy for diagnostics, without the need for advanced platforms.

**Figure 2:**
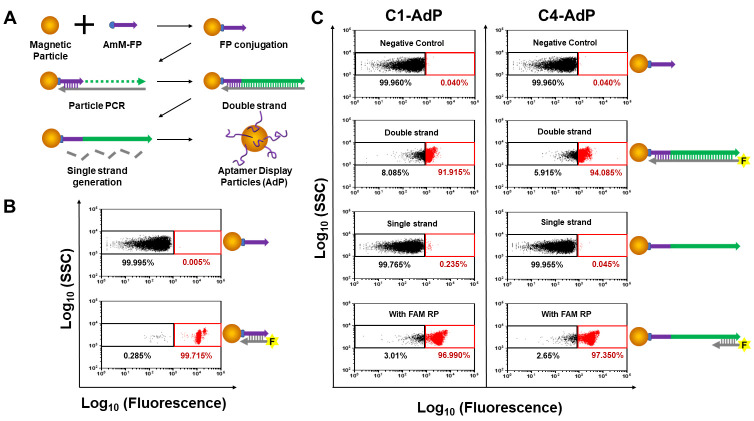
Process and schematic for generating AdP. (**A**) Procedure of generating AdP. Conjugating the AmM-FP on the magnetic beads using EDC/NHS chemistry to generate FP bead. The unique aptamer sequence is amplified on the FP bead by particle PCR. After PCR amplification, single-strand generation is performed by alkaline denaturation to remove the antisense strand. (**B**) Quantification of the FP conjugation. To confirm the conjugation efficiency, the FACS analysis was performed by the hybridization of the FAM-FPc. The black box indicates the “reference gate” that represents the fluorescence intensity of FP-coated particles without FAM-FPc hybridization as a negative control. (**C**) FACS dot plots from each procedure for generating SpS1-AdPs. The fluorescence intensities were quantified portions of the population shift to the red box from the reference gate.

The AdP was fabricated by a simple approach using PCR-based processes (Figure 2A). First, the amino-modified forward primer (AmM-FP) was conjugated to carboxylic acid-functionalized magnetic beads via carbodiimide crosslinking. The efficiency of FP conjugation was evaluated using FACS dot plotting with hybridization to the FAM-labeled forward primer complementary strand (FAM-FPc), which resulted in a population shift of over 99.7% (Figure 2B). Next, we performed particle PCR to generate the AdP using SpS1-C1 or SpS1-C4 aptamers as templates. This unique aptamer was fully displayed on the surface of the magnetic particles in double-stranded antisense strands. To confirm the efficiency of aptamer functionalization using particle PCR, we introduced FAM modification at the 5 ′ of the reverse primer (FAM-RP), which extended to the antisense strand with the aptamer sequence. The results showed a population shift of >91% in fluorescence from the reference gate (Figure 2C). To enable binding of the DNA aptamer, single-strand generation was performed by alkaline denaturation, allowing the release of antisense strands from the AdP. Subsequently, we confirmed that the fluorescence shift turned down to the reference gate. For further investigation, the AdP was hybridized with FAM-RP to evaluate whether the aptamer sequence on the particles was damaged by alkaline denaturation. Complete fluorescence recovery was observed, indicating that only antisense strands were released from the dsDNA. Using a simple process, we successfully generated spike protein-binding aptamer display particles (SpS1-AdPs), SpS1-C1 aptamer display particles (C1-AdP) and SpS1-C4 aptamer display particles (C4-AdP), for the sensitive detection of SARS-CoV-2. 

### 3.2. Accessing Binding Ability of SpS1-AdPs against SARS-CoV-2 Spike Protein

The surface protein, also known as the spike protein, is present on the exterior of SARS-CoV-2. It plays a crucial role in facilitating viral entry into host cells by interacting with human angiotensin-converting enzyme 2 (ACE2). In contrast to other proteins found in SARS-CoV-2, such as the nucleocapsid (N), membrane (M), and envelope (E) proteins, the spike protein is the primary antigen that can be employed in COVID-19 diagnostics. The two aptamers used in this study were screened against spike protein to allow rapid detection without virus lysis.

To evaluate the accessibility and detection efficacy of SpS1-AdPs for SARS-CoV-2, we conducted a binding assay using C1-AdP and C4-AdP with a recombinant spike protein in its pre-fusion state as a homotrimer [32]. First, biotinylation was performed on the primary amines presented by lysine groups on the spike protein using NHS-PEG_4_-Botin. Upon complex formation between SpS1-AdPs and biotinylated spike proteins, fluorescence signals were generated through interaction with Alexa Fluor 488-conjugated streptavidin. To confirm the fluorescence labeling, we devised a sandwich-type fluorescence assay utilizing streptavidin-coated magnetic particles and Alexa Fluor 488-conjugated streptavidin. The biotinylated spike protein was bound to streptavidin-coated particles and incubated with Alexa Fluor 488-conjugated streptavidin, followed by the FACS analysis after washing. A notable 99.7% population shift in fluorescence was observed, indicating successful biotinylation of the lysine residues on the spike proteins (Supplementary Information, Figure S1). Furthermore, to verify whether biotinylation influenced the affinity and specificity of aptamer for the spike protein, a binding assay and specificity test were carried out using SpS1-AdPs with spike protein, bovine serum albumin (BSA), and lysozyme under identical biotin molar excesses and concentrations (Supplementary Information, Figure S2).

**Figure 3:**
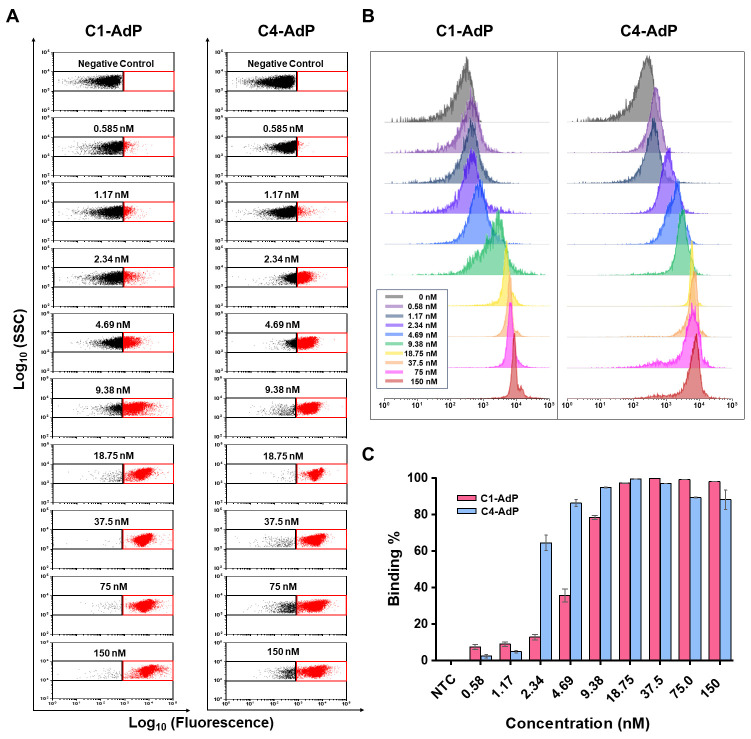
Evaluation of the binding performance of SARS-CoV-2 spike protein-binding AdPs by bead-based fluorescent assay. FACS dot plots (**A**) and histogram (**B**) from C1-AdP and C4 AdP, respectively, with the stated concentrations of biotinylated trimeric spike protein. The fluorescence signals were labeled by Alexa Flour 488-conjugated streptavidin, which binds the biotinylated spike protein after complexation with the SpS1-AdPs. (**C**) Fluorescence population shift from the reference gate quantified as percentages.

Next, we performed a bead-based fluorescence assay [33] using various concentrations of biotinylated spike proteins ranging from 0 to 150 nM. The results showed that C1-AdP and C4-AdP had high binding ability, with a concentration-dependent increase in fluorescence. Both SpS1-AdPs showed significant population shifts in fluorescence that were saturated at approximately 10 nM (Figure 3). In terms of the sensing ability of both SpS1-AdPs, a broad range of serially diluted spike proteins was successfully detected, even at low concentrations (0.5 nM). In summary, we showed the binding ability of our SpS1-AdPs to the SARS-CoV-2 spike protein and demonstrated their potential for the detection of live viruses.

### 3.3. Fluorometric Assay of SARS-CoV-2 Pseudoviruses Using SpS1-AdPs

**Figure 4:**
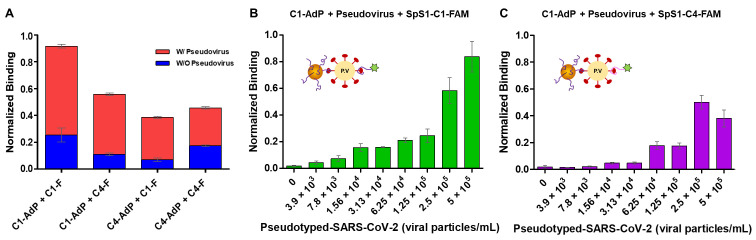
Sandwich-type fluorometric SARS-CoV-2 pseudovirus assay using SpS1-AdPs. (**A**) Comparison of the ideal combination of sandwich-type fluorometric assay with C1-AdP or C4-AdP as primary capture probes and FAM-modified SpS1-C1 and SpS1-C4 aptamers as secondary detection probes. Relative binding was calculated using a constant concentration of pseudovirus (5 × 10^5^ pseudoviral particles/mL). (**B**,**C**) The data were quantified on the fluorescence population shift using FACS dot plots, and the error bars correspond to the standard deviation of the triplicate experiments. The presented data were normalized to rescale between 0 and 1 using the following formula: (I − I_min_)/(I_max_ − I_min_).

Next, we explored whether we could use the SpS1-AdPs to detect SARS-CoV-2 using D614G-spike pseudoviral particles. A sandwich-type fluorometric assay was conducted using C1-AdP or C4-AdP as capture probes and fluorescent-labeled SpS1-C1 or SpS1-C4 aptamers as detection probes (Figure 4). As mentioned above, spike proteins are abundantly expressed on the outer membrane, and the pseudovirus mimics them on the surface.

Each of SpS1-AdPs was incubated with SARS-CoV-2 pseudoviruses for 1 h, followed by binding of the FAM-modified secondary aptamer bound for 1 h. We observed that combining C1-AdP as the primary probe and the FAM-SpS1-C1 aptamer as the secondary probe showed the highest percentage of fluorescence population shift in the FACS dot plot (Figure 4A). Considering the binding affinities of two aptamers, with Kd values of 1.47 nM and 1.81 nM for SpS1-C1 and SpS1-C4, respectively, the SpS1-C1 aptamer clearly exhibited higher binding levels even with pseudovirus. Other combinations of C1-C4, C4-C1, and C4-C4 were also able to detect pseudoviruses, compared with the negative control without the pseudovirus. For further investigation, C1-AdP was incubated with serially diluted pseudoviruses ranging from 10^5^ to 10^1^ pseudoviral particles/mL. After binding, the FAM-modified SpS1-C1 and SpS1-C4 aptamers were added, respectively, as secondary detection probes. The sandwich-type fluorometric SARS-CoV-2 pseudovirus assay showed a concentration-dependent increase in fluorescence. These data were simply interpreted via a population shift in fluorescence intensities compared with a reference gate. We determined the detection limit of our sensing system using an assay with various numbers of pseudoviral particles (Figure 4B,C). The fluorometric-based approach demonstrated a detection limit of 3.9 × 10^3^ pseudoviral particles/mL of SARS-CoV-2.

### 3.4. Colorimetric Assay of SARS-CoV-2 Pseudoviruses Using SpS1-AdPs

To improve the sensitivity of AdP-based detection methods, we employed a colorimetric assay with a signal amplification method. Nucleic acid aptamers have the greatest benefit in that the chemical synthesis of the sequence allows various types of modifications. Therefore, we introduced biotin modification on the 5′end of the secondary aptamer to allow conjugation with the avidin–HRP complex, resulting in the signal amplification of the catalytical oxidation of TMB by HRP.

**Figure 5:**
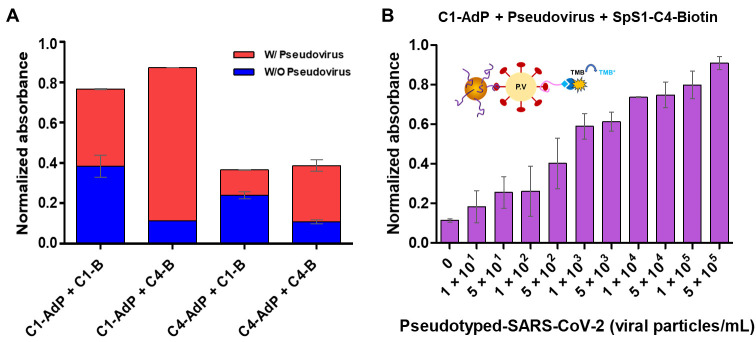
Sandwich-type colorimetric SARS-CoV-2 pseudovirus assay using SpS1-AdPs. (**A**) Assessment of the ideal combination of sandwich-type colorimetric assay with C1-AdP or C4-AdP as primary capture probes and biotin-modified SpS1-C1 and SpS1-C4 aptamer as secondary detection probes. The relative binding was calculated using a constant pseudovirus concentration (5 × 10^5^ pseudoviral particles/mL). The blue and red colors indicate background and positive signals, respectively. (**B**) The sandwich-type colorimetric assay was performed using C1-AdP as a primary capture probe and biotin-modified SpS1-C4 aptamer as a secondary detection probe to detect various numbers of pseudoviral particles. After the sandwich formation, avidin and HRP were introduced for signal amplification. The absorbance data were quantified at 450 nm, and the error bars correspond to the standard deviation of triplicate experiments. The presented data were normalized to rescale between 0 and 1 using the following formula: (A − A_min_)/(A_max_ − A_min_).

The sandwich-type colorimetric SARS-CoV-2 pseudovirus assay was performed under identical conditions as the fluorometric assay described above. Two SpS1-AdPs were used as primary capture probes and biotinylated SpS1-C1 and SpS1-C4 aptamers were used as detection probes. Prior to determining the detection limit using this approach, we compared the combination that was desired to satisfy the requirements of sensing a low background signal and high sensitivity. The combination of C1-AdP and SpS1-C1, which was the best in the fluorometric assay, resulted in higher background signals, despite having the highest positive signal (Figure 5A). High noise signals caused false-positive results that were difficult to interpret, leading to reduced sensor accuracy. In contrast, the combination of C1-AdP and SpS1-C4 showed ideal results with high positive and low background signals, even under identical conditions.

We further investigated the reason for the higher background signals in the C1-C1 combination. C1-AdP and C1-FAM, and C1-AdP and C4-FAM were assayed using a fluorometric assay without pseudovirus as a negative control (Figure 4). Compared with the FACS dot plots down to the decimal level, the C1-C1 combination had a slightly higher non-specific binding (0.343%) than the C1-C4 combination (0.051%) (Supplementary Information, Figure S3). Although the signals were negligible in the fluorometric assay, the signal amplification approach in the colorimetric assay could be affected by non-specific binders amplifying the background signals.

To determine the sensitivity of the colorimetric assay using a signal amplification approach, a combination of C1-AdP and SpS1-C4-biotin was used to detect different numbers of pseudoviral particles (10^5^–10^1^) (Figure 5B). The sandwich-type colorimetric assay using our high-quality aptamers achieved high sensitivity, with a detection limit of 1 × 10^1^ pseudoviral particles/mL. To summarize, our AdP-based colorimetric assay, specifically the C1-C4 combination, showed sensitive detection of SARS-CoV-2 pseudovirus over four orders of magnitude (10^5^–10^1^ pseudoviral particles/mL). Given these results with a broad detection range and low detection limit, our assay demonstrates competitive sensitivity even without advanced sensing platforms, compared to other colorimetric assays for SARS-CoV-2 detection reported in the literature [34]. These results indicate that our approach using high-quality SpS1-AdPs have the potential for the early detection of SARS-CoV-2.

## 4. Conclusions

We reported an aptamer-based sensing method to develop a diagnostic tool for SARS-CoV-2 that had the advantages of simple fabrication, ease of interpretation, and cost-effectiveness. Using high-quality aptamers against the SARS-CoV-2 spike protein, we showed unique aptamer display particles (AdP), leading to the precise analysis of individual particles using flow cytometry. Our SARS-CoV-2 spike protein-binding AdPs (SpS1-AdPs) showed high binding ability even at low concentrations (0.5 nM) when using the recombinant trimeric spike protein. Furthermore, we designed a sandwich-type SARS-CoV-2 pseudovirus assay to improve the selectivity and sensitivity using SpS1-AdPs as primary capture probes and signal-labeled SpS1-C1 or SpS1-C4 aptamers as secondary detection probes. In the flow cytometry-based fluorometric assay, the combination of C1-AdP and the SpS1-C1 aptamer successfully detected SARS-CoV-2 pseudovirus with a detection limit of 3.9 × 10^3^ pseudoviral particles/mL. To improve the sensitivity of the assay, an amplification approach using the catalytic oxidation of TMB by HRP was employed in the colorimetric assay, resulting in higher sensitivity, with a detection limit of 1 × 10^1^ pseudoviral particles/mL and a broad range of over four orders of magnitude (10^5^ to 10^1^ pseudoviral particles/mL). Our high-quality SpS1-AdPs successfully completed the sensitive detection of SARS-CoV-2, even without advanced platforms. We believe that our method can contribute to the generalization of aptamer-based sensing applications using popular equipment, such as flow cytometry and spectrometry.

## Data Availability

Data are contained within the article.

## Supplementary Materials

The following supporting information can be downloaded at: https://www.mdpi.com/article/10.3390/bios14030113/s1, Table S1: Primer sequences used in this study, Table S2: Two novel DNA aptamers against SARS-CoV-2 spike protein, Figure S1: Evaluation of the biotinylation on the SARS-CoV-2 spike protein, Figure S2: Specificity test of SARS-CoV-2 spike protein-binding aptamers, Figure S3: Assessment of non-specific binding between two spike protein-binding aptamers.
