# Supplementary material for: Fluorometric and Colorimetric Method for SARS-CoV-2 Detection Using Designed Aptamer Display Particles
Source: Biosensors (Basel). 2024 Feb 20;14(3):113. doi: 10.3390/bios14030113 (PMC10968392; doi:10.3390/bios14030113)
Supplement: Supplementary file 1 [file biosensors-14-00113-s001.zip › biosensors-2836664-supplementary.pdf]

biosensors

Supplementary Information

Fluorometric and Colorimetric Method for SARS-CoV-2 Detection Using Designed Aptamer Display Particles

Ki Sung Park †, Anna Choi †, Tae-In Park and Seung Pil Pack *

Department of Biotechnology and Bioinformatics, Korea University, Sejong 30019, Republic of Korea
†These authors contributed equally.
* Correspondence: author: spack@korea.ac.kr

Table S1. Primer sequences used in this study.

Name	Sequence (5\` to 3\` and N to C terminus)	Description
**FP**	GAACATTGGCGTCCGTGAG	Forward primer for PCR amplification
**RP**	CACTTCCTCAAACGCCCAA	Reverse primer for PCR amplification
**AmM-FP**	NH_2-GAACATTGGCGTCCGTGAG	Amino-modified forward primer for conjugation with carboxylic acid coated magnetic bead
**FAM-FPc**	FAM-CTCACGGACGCCAATGTTC	FAM modified forward primer complementary strand
**FAM-RP**	FAM-CACTTCCTCAAACGCCCAA	FAM modified reverse primer

Table S2. Two novel DNA aptamers against SARS-CoV-2 spike protein.

Aptamer ID	Sequence	Length	SELEX	Rounds	Dissociation Constant (*Kd*)
SpS1-C1	gaacattggcgtccgtgag-TGAGACCA-TAGTCCAGCGAACTAAAC-CTACCCTAAAGGG-CAAGGAAGACGGG-cacttcctcaaac-gcccaa*	90 mer	Particle display	4	1.47 ± 0.30 nM
SpS1-C4	gaacattggcgtccgtgag-CAGCTCGTGGTTGTTT-GCTTGTATACTTTT-GTGGTTTATCTT-GTTTCTGAT-cacttcctcaaacgcccaa	89 mer	Particle display	4	1.81 ± 0.39 nM

* Lowercase letters represent the sequences of forward primer and reverse primer for PCR amplification.

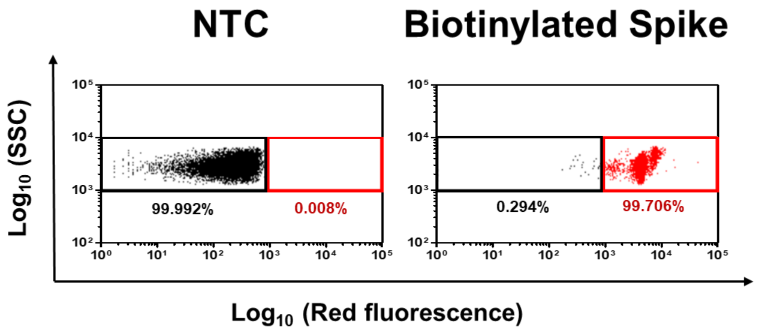

Figure S1. Evaluation of the biotinylation on the SARS-CoV-2 spike protein. We designed a sandwich-type fluorescence assay for the biotinylated spike protein using a streptavidin-coated magnetic bead and Alexa Flour 488 conjugated-streptavidin. FACS dot plots from negative control that used naïve spike protein (NTC) and positive control that sandwich formation with biotinylated spike protein (Biotinylated Spike). The black box represents a reference gate to compare with negative control, and the red box indicates fluorescence signals increased.

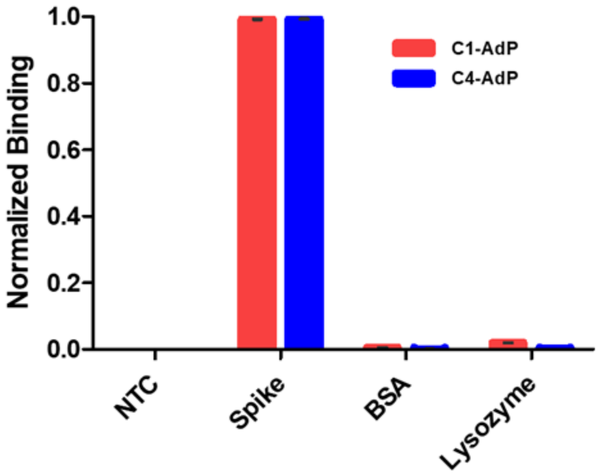

Figure S2. Specificity test of SARS-CoV-2 spike protein-binding aptamers. The specificity was also verified using the trimeric spike protein (Spike), bovine serum albumin (BSA), and Lysozyme. All proteins were biotinylated under identical conditions and molar excess of biotin. NTC indicates the background signal by non-specific binding as a negative control between only SpS1-AdPsand Alexa Flour 488-conjugated streptavidin. The presented data were normalized to rescale between 0 and 1 using the formula: $(I-I_{min})/(I_{max}-I_{min})$.

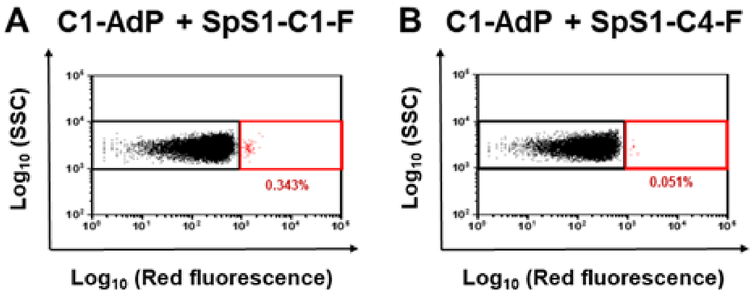

Figure S3. Assessment of non-specific binding between two spike protein-binding aptamers. FACS dot plots from non-specific interaction between SpS1-C1 aptamer display particles (C1-AdP) and FAM-modified SpS1-C1 (A) or SpS1-C4 (B) aptamer, respectively.
